# The paradox of eGFR trends and kidney failure incidence in patients without monogenic kidney disorders

**DOI:** 10.1172/JCI187470

**Published:** 2024-12-16

**Authors:** Xiaona Wang, Dongyan Wang

**Affiliations:** Department of Nephrology, Affiliated Hospital of Shandong University of Traditional Chinese Medicine, Jinan, Shandong, China.

**Keywords:** Nephrology, Urology

**To the Editor:** MD Elliott et al. showed increased risk of kidney failure in patients with genetic kidney disorders (1). The paper holds important clinical implications, as an increasing body of research indicates that the prevalence of monogenic genetic diseases in adults may be underestimated (2). In the “Study Design and Cohorts” section, the authors indicated that participants with diabetes mellitus were excluded. Nevertheless, the matching adjusted model employed for the analysis of the CureGN cohort data incorporated “diabetes” as a variable in the analysis. Figure 1D illustrates a change in eGFR of 0.25 ml/min/year in the no monogenic group, whereas Figure 1A depicts an increase in the cumulative incidence of kidney failure in the no monogenic group. Given the positive trend in eGFR, it is perplexing that the incidence of kidney failure continues to escalate annually.

## Version 1 12/16/2024

Electronic publication
